# Different Crystallization Behavior of Amorphous ITO Film by Rapid Infrared Annealing and Conventional Furnace Annealing Technology

**DOI:** 10.3390/ma16103803

**Published:** 2023-05-18

**Authors:** Jiaming Li, Liangbao Jiang, Xiaoyu Li, Junjie Luo, Jiaxi Liu, Minbo Wang, Yue Yan

**Affiliations:** 1 Baimtec Material Co., Ltd., Beijing Institute of Aeronautical Materials, Beijing 100095, China; limuzi3l@163.com (Jiaming Li); lixiaoyu621@126.com (X.L.);; 2 Beijing Engineering Research Center of Advanced Structural Transparencies to the Modern Traffic System, Beijing 100095, China

**Keywords:** RIA, ITO film, annealing, crystallization, holding time

## Abstract

An amorphous indium tin oxide (ITO) film (Ar/O_2_ = 80:0.5) was heated to 400 °C and maintained for 1–9 min using rapid infrared annealing (RIA) technology and conventional furnace annealing (CFA) technology. The effect of holding time on the structure, optical and electrical properties, and crystallization kinetics of ITO films, and on the mechanical properties of the chemically strengthened glass substrates, were revealed. The results show that the nucleation rate of ITO films produced by RIA is higher and the grain size is smaller than for CFA. When the RIA holding time exceeds 5 min, the sheet resistance of the ITO film is basically stable (8.75 Ω/sq). The effect of holding time on the mechanical properties of chemically strengthened glass substrates annealed using RIA technology is less than that of CFA technology. The percentage of compressive-stress decline of the strengthened glass after annealing using RIA technology is only 12–15% of that using CFA technology. For improving the optical and electrical properties of the amorphous ITO thin films, and the mechanical properties of the chemically strengthened glass substrates, RIA technology is more efficient than CFA technology.

## 1. Introduction

Transparent conductive oxide films are widely used, for example, indium tin oxide (ITO) is used in liquid crystal displays [1], electrochromic windows [2], solar cells [3], electrical-heated glass [4] for aircraft (anti-ice defogging), and so on. ITO is an n-type semiconductor film with low resistivity (10^−4^–10^−3^ Ω·cm) [5]. The properties of ITO films mainly depend on the properties of the substrate [6], preparation method [7] and deposition process parameters (including oxygen partial pressure [8,9] and substrate temperature [10]), and heat treatment of the film [11]. An improved preparation process is needed to prepare films with low resistivity, high transmittance, and smooth surface morphology. For a thin film, transmittance and conductivity are two contradictory properties. To obtain a thin film with high transmittance and high conductivity, a crystalline film is generally directly prepared by a high-temperature deposition method, or an amorphous film is deposited at room temperature and then crystallized by conventional furnace annealing (CFA) [12]. For some substrate materials that are not resistant to high temperature, high-quality films cannot be obtained by high-temperature deposition, and can only be crystallized by post-annealing. During the crystallization process of post-annealing, the properties of a film and substrate will change [13,14]. To better understand the influence of annealing on the crystallization behavior of a film, in addition to crystallization temperature [15], it is also necessary to study the influence of annealing technology and holding time on the properties of a film and its substrate.

In this paper, an amorphous ITO film was heated to 400 °C and maintained for 1–9 min using rapid infrared annealing (RIA) technology and CFA technology. The effects of holding time on the structure, optical properties, electrical properties, and crystallization kinetics of ITO films, and the mechanical properties of the chemically strengthened glass substrate, were revealed. The results showed that the crystallinity of the ITO film, annealed by the two techniques, was higher after being kept at 400 °C for 1–9 min. At 400 °C, with an increase in the holding time, the size of the surface clusters of the film did not change significantly, and the consistency of the surface morphology improved. When using RIA, the nucleation rate of ITO films was higher and the grain size was smaller; while the nucleation rate of ITO films produced using CFA technology was lower, the grain size was larger, and the size distribution was not uniform. In addition, the sheet resistance of the ITO film produced using CFA technology was slightly smaller than that of the film produced using RIA technology. When RIA technology was used for annealing, and the holding time was more than 5 min, the sheet resistance of the ITO film was basically stable (8.75 Ω/sq). The effect of holding time on the mechanical properties of chemically strengthened glass substrates annealed using RIA technology was less than that annealed using CFA technology.

## 2. Materials and Methods

### 2.1. Deposition of Amorphous ITO Films on Chemically Strengthened Glass Substrates

Amorphous ITO films (average thickness 330 nm ± 5 nm) were sputtered on chemically strengthened glass substrates (300 mm × 300 mm × 1.8 mm) using DC magnetron sputtering technology (Equipment model: JCP500, Techno, Beijing, China) at room temperature. A detailed description of the preparation of the chemically strengthened glass substrates can be found in the literature [16]. The distance from the ITO target (10 wt.% SnO_2_, 90 mm diameter, 99.99% purity) to the glass substrate was 90 mm, and the DC power supply voltage was 3.0 kW. During sputtering, the vacuum of the background was 8.0 × 10^−5^ Torr. The working gas was argon, with a flow of 80 sccm, and the reacting gas introduced was oxygen, with a flow of 0.5 sccm. The working gas pressure was 5.0 × 10^−3^ Torr which was controlled by the pressure controller.

### 2.2. Annealing

In this paper, the argon–oxygen ratio (Ar/O_2_) of the amorphous ITO film was 80 sccm:0.5 sccm, and the ITO glass was annealed using RIA and CFA technology under vacuum atmosphere, with a heating rate of 100 °C/min and 10 °C/min, respectively, to a temperature of 400 °C, maintained for 1–9 min, and then cooled to room temperature within the furnace. The infrared heated radiation lamp had a wavelength of 2.0 μm and a power of 1200 W. The model of the CFA equipment used in the experiment was CVD-100 (Techno, Beijing, China).

### 2.3. Characterization

In this paper, a surface profilometer (P-7, KLA Tencor, Silicon Valley, CA, USA) was used to measure the average film thickness. An X-ray diffractometer (Smart Lab, Tokyo, Japan) was used to characterize and analyze film structures. Kα radiation of Cu was used as the radiation source, the X-ray tube voltage was 40.0 kV, and the tube current was 150.0 mA. The grazing incidence angle was 5°, the scanning range was 10°–90°, the scanning speed was 8°/min, and the sampling interval was 0.02°. Cold-field emission scanning electron microscopy (SU-8010, Hitachi, Tokyo, Japan) was used to obtain the surface micro-morphology and cross-sectional morphology of the films before and after crystallization. The voltage of the scanning electron microscope was set to 3.0–5.0 kV, and the current was 10.0 µA. The cross-sectional sample (10 mm × 10 mm × 1.8 mm) was a polished, coated glass cross-section. The prepared sample with a clear cross-sectional structure was placed on the SEM equipment test sample table for testing. Atomic force microscopy was used to measure and analyze the root mean square surface roughness of the film before and after crystallization. The tapping scanning mode was selected, the scanning frequency was 1 Hz, and the scanning range was 10 µm. A four-point probe (SD-800, NAGY, Berlin, Germany) was used to measure the sheet resistance of the films. The normal temperature test module of the Hall Effect System (HL5500, Nano metrics, Katana, ON, Canada) was used to measure the carrier concentration, carrier mobility, and resistivity of the ITO films before and after annealing. The transmittance of ITO films before and after annealing was measured using a UV-Vis-NIR spectrophotometer (5000, Varian, Palo Alto, CA, USA). Before and after the annealing process, a surface stress meter (FSM-6000LE, ORIHARA, Osaka, Japan) was used to measure the surface compressive stress and stress layer depth of the chemically strengthened glass, with a stress resolution of 1 MPa and a stress layer depth resolution of 0.1 µm.

## 3. Results and Discussion

### 3.1. Structural Analysis

**Figure 1:**
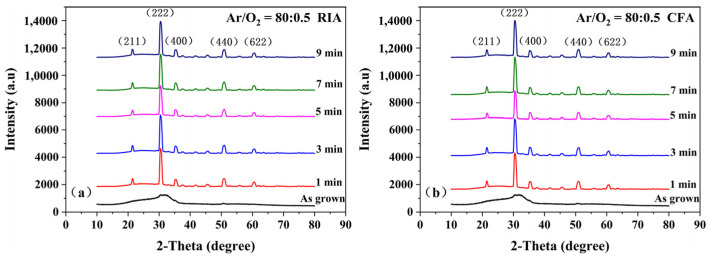
X-ray diffraction pattern of a film (**a**) annealed by rapid infrared annealing (RIA) and (**b**) annealed by conventional furnace annealing (CFA) with different holding time.

The X-ray diffraction patterns of amorphous ITO films, before and after annealing with different holding times, are shown in Figure 1. It can be seen from Figure 1 that all diffraction peaks of the films, produced by both RIA and CFA, correspond to the In_2_O_3_ characteristic peaks (211), (222), (400), (440), and (622) of the cubic phase, and there are no diffraction peaks of Sn and Sn compounds, indicating that Sn atoms are completely substituted in the In_2_O_3_ lattice, forming a uniform solid solution. The ITO films deposited at room temperature are amorphous. After annealing at 400 °C and holding for 1–9 min, it is found that all ITO films have characteristic diffraction peaks, and the intensity of the (222) peak is higher, indicating that the ITO films have a high degree of crystallinity [17]. With an increase in holding time, the crystal growth is mainly oriented with the (222) plane, but the intensity of the (222) peak does not change significantly, indicating that the ITO film has a high degree of preferred orientation at this annealing temperature. The structural change trend of the ITO film produced by RIA is consistent with that by CFA, and there is no obvious difference. It can be seen from the literature [15] that annealing temperature has a greater effect on the structure of ITO films than the holding time, which has a lesser effect when an appropriate annealing temperature is selected. The results displayed by the XRD spectrum of ITO may be related to cluster formation and surface atomic arrangement, such as surface plasmons, etc [18].

According to the XRD pattern, the grain size *D* can be calculated using the Debye–Scherrer formula [19]:

(1) $$D=\frac{0.94\lambda }{\beta \cos\theta },$$

where *λ* is the X-ray wavelength (*λ* = 0.154050 nm), *β* is the full width at half maximum in radian, and *θ* is Bragg’s angle.

The angle of preferred orientation of (222), the grain size of the (222) plane, and the calculated results of average grain size of the ITO film, after annealing with different holding times, are shown in Table 1.

It can be seen that the average grain size is in the range of 13–15 nm. The 2θ angle corresponding to the main (222) peak shifted with prolonged holding time, which may be related to lattice distortion caused by the substitution of In^3+^ ions (0.079 nm) by Sn^4+^ ions (0.069 nm) or Sn^2+^ ions (0.093 nm) [20]. In addition, the 2θ angle corresponding to the (222) peak of the ITO film obtained using RIA technology is slightly smaller than that obtained by CFA. The 2θ angle corresponding to the (222) peak becomes smaller, which may be related to the stress of the film [21]. The heating and cooling rates of RIA are faster, and the impact on the film stress is greater than that of CFA.

### 3.2. Morphological Analysis

**Figure 2:**
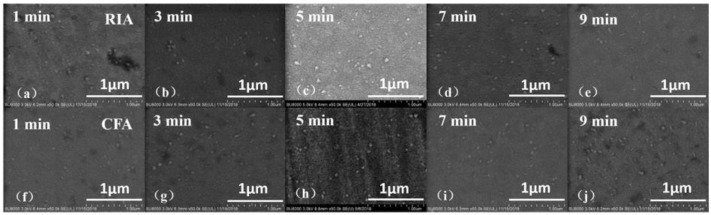
SEM image of the surface morphology of the ITO film (**a**–**e**) annealed using RIA, holding for 1–9 min (**f**–**j**) annealed using CFA, holding for 1–9 min.

The crystallization of ITO thin films is a process of transition from an amorphous state to a crystalline state. During this process, the microscopic morphology of the thin film will change, which in turn affects the properties of the ITO thin film. Figure 2 shows the surface morphology of an amorphous ITO film after RIA and CFA. Figure 2a–e shows the surface microstructure of an ITO film after RIA, heating to 400 °C and holding for 1–9 min. Figure 2f–j shows the surface microstructure of an ITO film after CFA, heating to 400 °C and holding for 1–9 min. Figure 2 shows that some clusters are formed on the surface of the film, and the average size of the clusters is found to be in the range of 52–59 nm after measurement. When the temperature is maintained at 400 °C for 1–9 min, changing the holding time has little effect on the surface morphology of the ITO film. At 400 °C, the change of cluster size on the surface of the film is not obvious, and the consistency of the surface morphology is improved. Table 2 shows the obtained cluster sizes. Due to the post-annealing method adopted in this paper, each atom of the amorphous ITO film has already occupied a fixed position in the lattice during the deposition process, and the post-annealing process allows the atoms to move only near the original position. In addition, the annealing in this paper is carried out in a vacuum environment; the interaction between atoms and oxygen is small, and the grains aggregate to form larger clusters [22]. Compared with the annealing temperature, the annealing holding time has less effect on the surface morphology of the ITO films [15].

**Figure 3:**
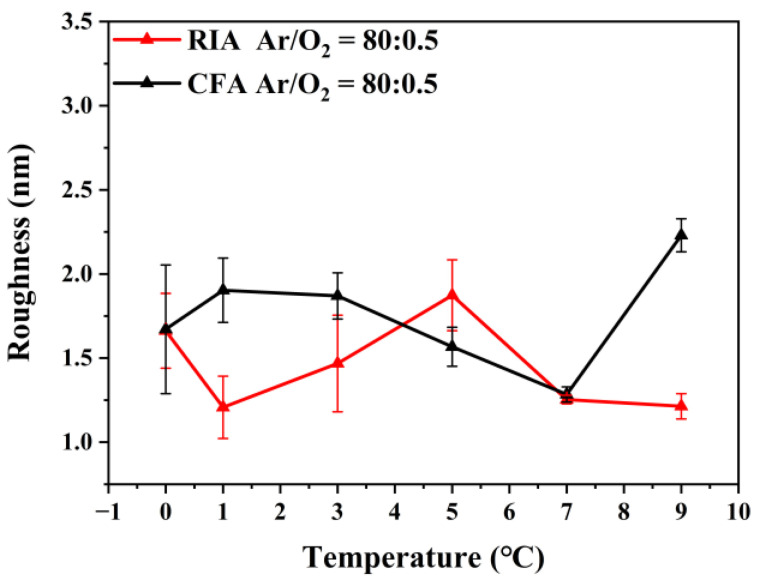
The root mean square roughness of the ITO film after annealing with different holding time.

To further study the effect of holding time on the surface morphology of ITO thin films, AFM was used to analyze the surface morphology of ITO films before and after crystallization. Figure 3 shows the root mean square roughness of the surface of ITO films after annealing with different holding times. It can be seen from Figure 3 that, with the increase of holding time, the roughness fluctuates in the range of 1.2–2.2 nm. When the amorphous ITO film is annealed using RIA technology at 400 °C, the surface roughness of the film first increases and then decreases with the increase of holding time. The maximum roughness of the ITO film obtained after holding for 5 min is 1.87 nm, and the roughness of the ITO thin film finally decreases to 1.21 nm with increasing holding time. When using CFA, with the increase of holding time, the surface roughness of the film first decreases and then increases.

**Figure 4:**
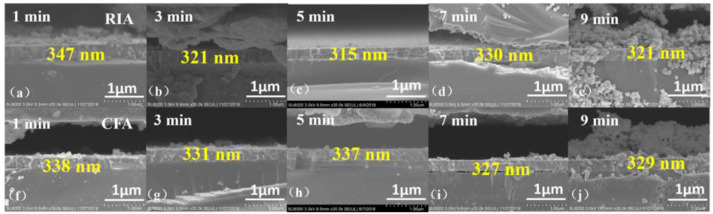
The cross-section morphology of the ITO film heated at 400 °C and held for 1–9 min, (**a**–**e**) RIA, (**f**–**j**) CFA.

Figure 4 shows the cross-sectional morphology of the ITO films after heating at 400 °C and holding for 1–9 min. Figure 4a–e is the micro-morphology of the cross-section after RIA, and Figure 4f–j the micro-morphology of the cross-section after CFA. It can be seen from Figure 4 that, when RIA technology is used, the average thickness of the film fluctuates with increasing holding time. Specifically, the average thickness of the ITO film decreases with an increase of holding time to 5 min. When the holding time is prolonged, the average thickness of the ITO film increases slightly, and finally decreases when the holding time is 9 min. However, compared with the amorphous ITO film, the annealed film becomes slightly denser, and this phenomenon occurs for both RIA and CFA.

Table 3 shows the average thickness of ITO films after annealing with different holding time. It can be seen from the table that when RIA technology is used, the average thickness of the film decreases from 347 nm to 315 nm with an increase of holding time from 1 min to 5 min. When the holding time increases to 7 min, the film thickness increases to 330 nm. The film thickness reduces to 321 nm after holding for 9 min. However, the average thickness of the ITO film fluctuates in the range of 327–338 nm when annealed using CFA. The increase in film thickness may be due to the reduction of film lattice defects during annealing, the increase of crystallinity of the ITO film, and the initiation of grain growth [23]. The results may be related to the issue of strain or displacement of grain boundaries [18]. The decrease in film thickness during annealing may be due to desorption of oxygen, i.e., that adsorbed during the deposition process and that in the interstitial space of the ITO film [24], resulting in an increase in the density of the ITO film cross-sectional structure.

### 3.3. Crystallization Kinetics Behavior Analysis

The process of ITO film transition from an amorphous state to a crystalline state is accompanied by nucleation and growth, and nucleation and growth influence each other [25]. During the crystallization process, not all nucleation points can nucleate, and only stable nuclei can crystallize and grow. When a crystal nucleus is formed, the free energy of the system is reduced, and the free energy of the interface is also increased due to the increase of the surface area.

During the crystallization process, different heating rates also affect the film nucleation behavior. During a rapid heating process, the surface temperature of the film can reach the surface critical nucleation temperature in a short time. The temperature difference between the film surface and the film–substrate interface is large, and nucleation can occur near the film surface and film–substrate interface, resulting in a high nucleation rate. In a slow heating process, nucleation and growth near the film–substrate interface occurs first; the surface temperature of the film cannot reach the critical nucleation temperature in a short time, as such nucleation occurs mainly at the film–substrate interface at this initial heating stage. Although the surface temperature of the film may satisfy the energy conditions for nucleation after heating for a certain time, the nucleation rate for a slow heating process is low, due to unsatisfactory nucleation at the interface of the film and substrate, and the material conditions on the surface of the growing film. It can be predicted that, at the same crystallization temperature, the grain size of the thin film is relatively small due to the high nucleation rate. However, the films with low nucleation rate are dominated by grain growth in the later heating process, resulting in larger grain size and uneven size distribution of the films.

**Figure 5:**
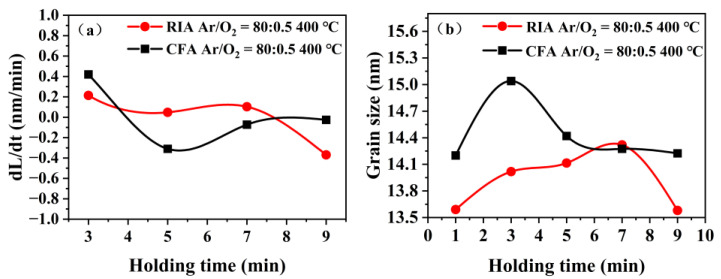
(**a**) Grain size change rate with different holding time (**b**) Grain size with different holding time of the ITO film (Ar/O_2_ = 80:0.5) annealed at 400 °C.

Figure 5 shows the rate of change in grain size and the grain size of an ITO film (Ar/O_2_ = 80:0.5) after annealing at 400 °C for different holding times. The rate of change in film grain size is defined as $V(L)=\frac{dL}{dt}$, where *L* is the grain size and *t* is the holding time. It can be seen from Figure 5a that the fluctuation rate range of the change in gain size of an ITO film after RIA (heating rate of 100 °C/min) is slightly smaller than that after CFA (heating rate of 10 °C/min). It can be seen from Figure 5b that the grain size of the ITO film after RIA is generally smaller than that after CFA. In addition, with the increase of holding time, the grain size of the ITO film first increases and then decreases. The trend of change in grain size of the film produced by RIA lags behind that of CFA. These experimental results are consistent with the analysis results of the film crystallization kinetics, that is, fast heating has the characteristics of high nucleation rate and small grain size in a thin film. However, slow heating leads to a low nucleation rate of the film, and the growth of crystal nuclei is dominant in the later stage, as such the grain size is large and the size distribution is not uniform.

### 3.4. Electrical Properties Analysis

**Figure 6:**
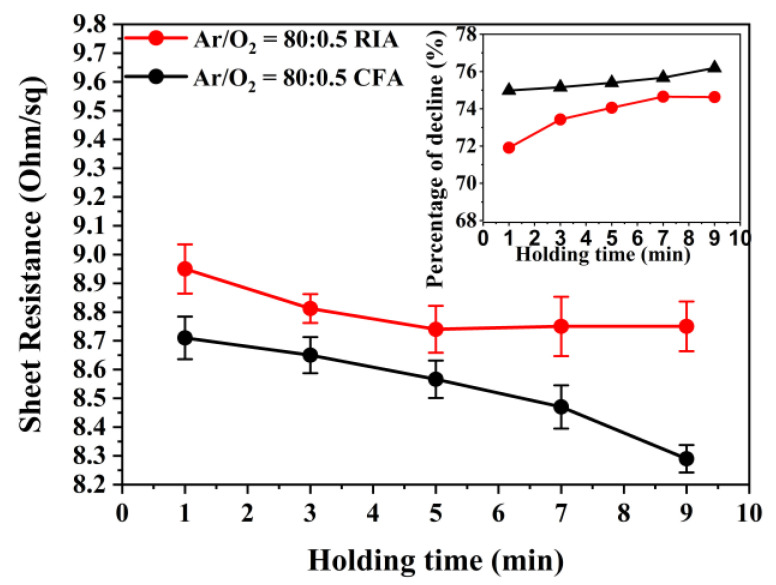
Sheet resistance of ITO film after annealing with different holding time (inset: the percentage of the decline in the sheet resistance).

Figure 6 shows the effect of holding time on the sheet resistance of ITO thin films (Ar/O_2_ = 80:0.5) after annealing. It can be seen from Figure 6 that, with an increase of the annealing holding time, the sheet resistance of the ITO film annealed using RIA technology decreases and the percentage of sheet-resistance decline increases. When the holding time of RIA increases from 1 min to 9 min, the sheet resistance of the ITO film decreases to 8.95 Ω/sq, 8.81 Ω/sq, 8.75 Ω/sq, 8.75 Ω/sq, and 8.75 Ω/sq, respectively. After CFA, the sheet resistance of the ITO film reduces to 8.71 Ω/sq, 8.65 Ω/sq, 8.56 Ω/sq, 8.47 Ω/sq, and 8.29 Ω/sq, respectively. After annealing for the same holding time, although the sheet resistance of the ITO film after CFA is slightly smaller than that of RIA, there is little difference between the two heat treatment techniques. When the annealing holding time is longer than 5 min with RIA, the sheet resistance of the ITO film is basically stable, indicating that a lower sheet resistance can be obtained by using this crystallization technology for 5 min when annealing at 400 °C. With CFA, the sheet resistance of the ITO film decreases slightly with an increase of the holding time, which may be related to the total time of the annealing process of this technology, and that the heat accumulation in the whole annealing process is greater than that using RIA technology. In addition, it is also possible that the grain size of the ITO film produced using CFA technology is slightly larger than that using RIA technology, which reduces the density of grain boundaries, reduces the scattering of carriers by grain boundaries, and improves the conductivity of the film.

**Figure 7:**
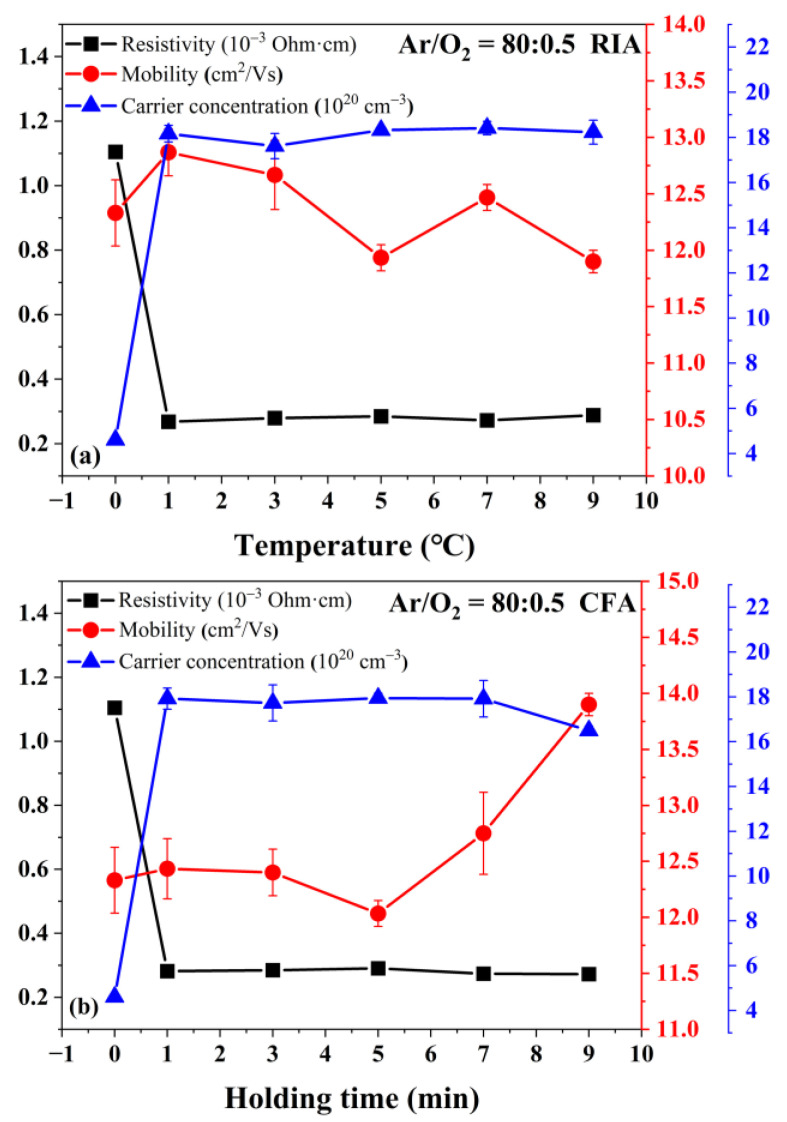
Effect of the holding time on the resistivity, mobility, and carrier concentration of the ITO film annealed using (**a**) RIA technology and (**b**) CFA technology.

Figure 7 shows the effect of holding time on resistivity, carrier mobility, and carrier concentration. It can be seen from Figure 7 that the resistivity of the film reduces from 1.1 × 10^−3^ Ohm·cm (amorphous) to 2.7 × 10^−4^ Ohm·cm when annealed at 400 °C for 1 min using RIA technology. When annealed at 400 °C using CFA technology, the resistivity of the film reduces from 1.1 × 10^−3^ Ohm·cm (amorphous) to 2.8 × 10^−4^ Ohm·cm after holding for 1 min. There is no significant difference in sheet resistivity with increasing holding time. After annealing for 1 min, the resistivity of the amorphous film decreases significantly, mainly due to the large increase in the carrier concentration [26]. During the annealing process, Sn^2+^ is oxidized to Sn^4+^ and replaces In^3+^ to provide electrons, resulting in an increase in carrier concentration [27]. During this process, the adsorbed oxygen and interstitial oxygen in the amorphous ITO film are desorbed, which increases the content of oxygen vacancies in the ITO film, and one oxygen vacancy can provide two free electrons and introduce a donor in the energy band structure [28].

### 3.5. Optical Properties Analysis

**Figure 8:**
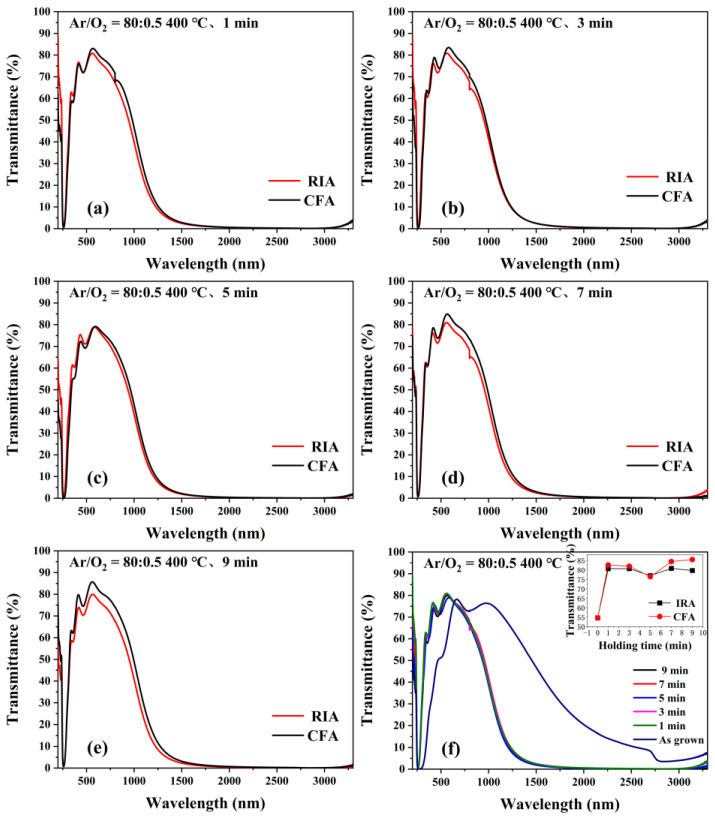
Transmittance spectrum of ITO thin films (**a**–**e**) using RIA technology and CFA technology with holding for 1–9 min. (**f**) transmittance spectrum of the ITO film annealed by RIA technology.

Figure 8 shows the transmittance spectra of ITO films obtained using RIA technology and CFA technology with holding for 1–9 min. It can be seen from Figure 8a–e that there is no obvious difference in transmittance of the ITO film produced using RIA technology or CFA technology. However, after annealing at 400 °C, with the increase of holding time, the transmission of the ITO film at 550 nm first decreases and then increases. The transmittance at 550 nm of the amorphous ITO film is 52.5%. After annealing at 400 °C for 1–9 min, the transmittance at 550 nm of the ITO film after RIA increases to 80.6%, 80.6%, 76.9%, 80.8%, and 79.6%, respectively. The transmittance at 550 nm of the ITO film after CFA increases to 82.6%, 81.9%, 76.4%, 84.3%, and 85.4%, respectively. The films annealed using CFA technology have a slightly higher transmittance, mainly because the whole process of annealing with this crystallization technology takes a long time, which will improve the crystallinity of the film, thereby improving the transmittance of the film [29]. Other peaks in the UV-vis spectra may be due to surface plasmon or non-spherically shaped crystallites [30].

**Figure 9:**
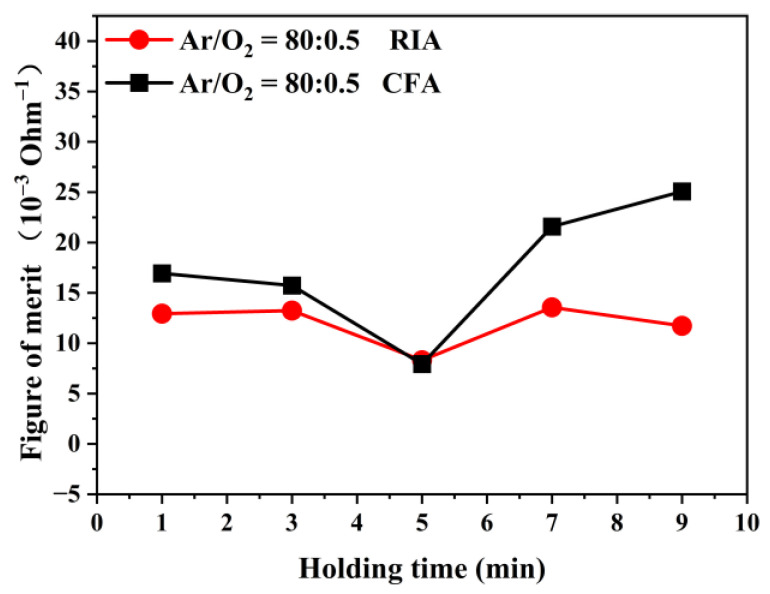
Quality factors of the amorphous ITO film after annealing for different holding time.

To study the effect of holding time on the optical and electrical properties of ITO thin films, a quality factor was calculated according to the Haacke equation [15]. Figure 9 shows the quality factor of amorphous ITO films after annealing for different holding times. It can be seen from Figure 9 that, when annealed using RIA technology, the quality factor of the films first decrease and then increase with an increase of holding time, and changes are in the range of 8.3 × 10^−3^ Ohm^−1^ to 13.5 × 10^−3^ Ohm^−1^. Using CFA technology, the film quality factor varies in the range of 7.9 × 10^−3^ Ohm^−1^ to 25.0 × 10^−3^ Ohm^−1^. When the holding time is longer than 7 min, the comprehensive optical and electrical properties of the ITO film produced using CFA technology are slightly higher than those using RIA technology, but the quality factors of the ITO film obtained after annealing by the two crystallization techniques are of the same order of magnitude.

**Figure 10:**
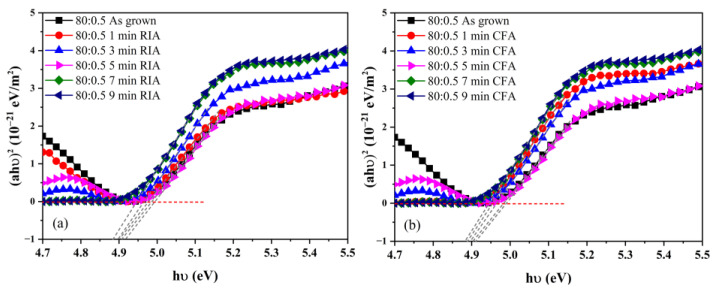
The dependence of the absorption coefficient on the photon energy for ITO thin films annealed by (**a**) RIA and (**b**) CFA for different holding time.

Figure 10 shows the dependence of the absorption coefficient on the photon energy for ITO films produced by RIA and CFA with different holding times. The optical bandgap (*Eg*) of the ITO films can be obtained by the extrapolation method and are shown in Table 4. From Figure 10 and Table 4, it can be determined that when an amorphous ITO film (Ar/O_2_ = 80:0.5) is annealed using RIA technology, the optical bandgap of the film changes in the range of 4.94–4.99 eV, and it first widens and then narrows with an increase in holding time. When annealed using CFA technology, the optical bandgap of the film changes in the range of 4.94–4.99 eV, and the trend is consistent with RIA technology. The widening of the optical bandgap may be due to an increase of the carrier concentration during the annealing process, which leads to an increase of the Fermi level entering the conduction band, and the Moss–Burstein effect appears [31]. The optical bandgap narrowing may be due to scattering between free carriers or between carriers and ion doping [32]. The optical bandgap of ITO films produced by RIA and CFA for the same holding time is basically the same.

### 3.6. Compressive Stress Analysis

**Figure 11:**
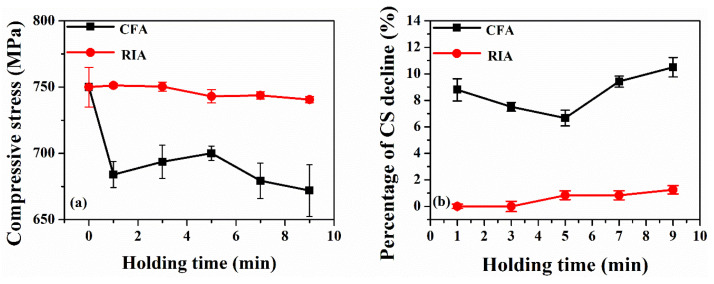
Mechanical properties of chemically strengthened glass with different holding times. (**a**) Compressive stress and (**b**) the percentage of glass compressive stress decline.

Figure 11 shows the effect of holding time on the mechanical properties of the chemically strengthened glass substrate. The figure shows that RIA technology can greatly reduce the attenuation of the glass mechanical properties during annealing. After the same annealing temperature and holding time, the compressive stress of the strengthened glass annealed using CFA technology is significantly lower than that using RIA technology. In addition, the mechanical properties of the glass substrate after annealing with different holding time using RIA technology have little difference, which is mainly due to the selection and optimization of the infrared wavelength of radiation used in this technology. By selecting a suitable wavelength, the photon energy absorption by the ITO film is increased, and at the same time, less energy is absorbed by the chemically strengthened glass, which reduces the temperature of the glass substrate. The surface compressive stress and compressive stress reduction percentage of glass after annealing are shown in Table 5.

## 4. Conclusions

In this paper, the effects of holding time on the structure, properties, and crystallization kinetics of ITO films (Ar/O_2_ = 80:0.5) and chemically strengthened glass were investigated. The results show that the holding time has little effect on the cluster size of the thin film surface, and the film surface morphology after annealing is more consistent. When annealed using RIA technology, the nucleation rate of ITO films is high, and the grain size is small. While the nucleation rate of ITO films is low when annealed using CFA technology, the grain size is large, and the size distribution is not uniform. After annealing at 400 °C and holding for 1–9 min, the sheet resistance of the ITO film obtained using CFA technology is slightly smaller than that using RIA technology. When the RIA annealing holding time exceeds 5 min, the sheet resistance of the ITO films is basically stable (8.75 Ω/sq). The effect of holding time on the mechanical properties of chemically strengthened glass substrates annealed using RIA technology is less than that using CFA technology. The percentage of the compressive stress decline of the glass after annealing using RIA technology is only 12–15% of that using CFA technology.

## Figures and Tables

**Table 1:** Grain size of the ITO film (Ar/O_2_ = 80:0.5) after annealing with different holding time.

Ar/O_2_	Holding Time (min)	RIA			CFA		
		2θ Angle of (222) Peak	Grain Size of (222) Peak (nm)	Average Grain Size (nm)	2θ Angle of (222) Peak	Grain Size of (222) Peak (nm)	Average Grain Size (nm)
80:0.5	1	30.36	13.29	13.59	30.41	13.39	14.20
	3	30.36	13.56	14.01	30.42	13.09	15.04
	5	30.34	13.27	14.11	30.32	12.91	14.42
	7	30.34	13.46	14.32	30.40	12.54	14.27
	9	30.34	13.39	13.58	30.36	11.18	14.22

**Table 2:** Average size of clusters on the ITO film surface after annealing for different holding time.

Holding Time	Crystallization Method	
	RIA	CFA
1 min	54.39 nm	54.16 nm
3 min	52.98 nm	58.75 nm
5 min	53.67 nm	52.00 nm
7 min	53.27 nm	53.02 nm
9 min	56.22 nm	54.95 nm

**Table 3:** Average thickness of the ITO film after annealing with different holding time.

	Holding Time	1 min	3 min	5 min	7 min	9 min
Technology						
RIA		347 nm	321 nm	315 nm	330 nm	321 nm
CFA		338 nm	331 nm	337 nm	327 nm	329 nm

**Table 4:** Optical bandgap of ITO films after annealing with different holding time.

Eg (eV)	Holding Time					
	Amorphous	1 min	3 min	5 min	7 min	9 min
RIA	4.98	4.97	4.96	4.99	4.94	4.94
CFA	4.98	4.96	4.96	4.99	4.94	4.94

**Table 5:** Mechanical properties of chemically strengthened glass after annealing with different holding time.

Holding Time (min)	RIA		CFA	
	Surface Compressive Stress (MPa)	Percentage of the Compressive Stress Decline (%)	Surface Compressive Stress (MPa)	Percentage of the Compressive Stress Decline (%)
1	751	0	684	8.8
3	750	0	693	7.6
5	743	0.9	700	6.7
7	743	0.9	679	9.4
9	740	1.3	671	10.5

## Data Availability

Not applicable.

## References

[1] Kim J., Lee D., Song S., Cho S.Y., Bae J.-S., Kim W., Youn B., Kim Y., Lee J.-S., Bu S.D. (2017). Surface chemistry modification in ITO films induced by Sn2+ ionic state variation. Curr. Appl. Phys..

[2] KLi K.D., Chen P.W., Chang K.S., Hsu S.C., Jan D.J. (2018). Indium-Zinc-Tin-Oxide film prepared by reactive magnetron sputtering for electrochromic ap-plications. Materials.

[3] Zhao S., Lv Z., Guo X., Liu C., Wang H., Jiang W., Liu S., Wang N., Cui Y., Ding W. (2018). The Diffusion of Low-Energy Methyl Group on ITO Film Surface and Its Impact on Optical-Electrical Properties. Materials.

[4] Szyszka B., Dewald W., Gurram S.K., Pflug A., Schulz C., Siemers M., Sittinger V., Ulrich S. (2012). Recent developments in the field of transparent conductive oxide films for spectral selective coatings, electronics and photovoltaics. Curr. Appl. Phys..

[5] Vilca-Huayhua C., Paz-Corrales K., Aragón F., Mathpal M., Villegas-Lelovsky L., Coaquira J., Pacheco-Salazar D. (2020). Growth and vacuum post-annealing effect on the structural, electrical and optical properties of Sn-doped In2O3 thin films. Thin Solid Film..

[6] Zhu H., Zhang H., Zhang T.-H., Yu S.-J., Guo P.-C., Wang Y.-X., Yang Z.-S. (2021). Optical and electrical properties of ITO film on flexible fluorphlogopite substrate. Ceram. Int..

[7] Hacini A., Ali A.H., Adnan N.N. (2021). Optimization of ITO thin film properties as a function of deposition time using the swanepoel method. Opt. Mater..

[8] Gwamuri J., Marikkannan M., Mayandi J., Bowen P.K., Pearce J.M. (2016). Influence of Oxygen Concentration on the Performance of Ultra-Thin RF Magnetron Sputter Deposited Indium Tin Oxide Films as a Top Electrode for Photovoltaic Devices. Materials.

[9] Marciel A., Graça M., Bastos A., Pereira L., Kumar J.S., Borges J., Vaz F., Peres M., Magalhães S., Lorenz K. (2021). Molybdenum Oxide Thin Films Grown on Flexible ITO-Coated PET Substrates. Materials.

[10] Mei-Zhen G., Ke X., Fahrner W.R. (2009). Study of the morphological change of amorphous ITO films after temperature-humidity treatment. J. Non-Cryst. Solids.

[11] Kaźmierczak-Bałata A., Bodzenta J., Dehbashi M., Mayandi J., Venkatachalapathy V. (2023). Influence of post processing on thermal conductivity of ITO thin films. Materials.

[12] Shi Z., Song L., Zhang T. (2017). Terahertz reflection and visible light transmission of ITO films affected by annealing temperature and applied in metamaterial absorber. Vacuum.

[13] Kurokawa T., Mori R., Norimasa O., Chiba T., Eguchi R., Takashiri M. (2020). Influences of substrate types and heat treatment conditions on structural and thermoelectric properties of nanocrystalline Bi_2_Te_3_ thin films formed by DC magnetron sputtering. Vacuum.

[14] Nandihalli N. (2022). Thermoelectric films and periodic structures and spin Seebeck effect systems: Facets of performance optimi-zation. Mater. Today Energy.

[15] Li J., Jiang L., Chen M., Li X., Wei Y., Ma Y., Fu Z., Yan Y. (2019). Structure and physical properties evolution of ITO film during amorphous-crystalline transition using a highly effective annealing technique. Ceram. Int..

[16] Li X., Jiang L., Wang Y., Mohagheghian I., Dear J.P., Li L., Yan Y. (2017). Correlation between K^+^-Na^+^ diffusion coefficient and flexural strength of chemically tempered aluminosilicate glass. J. Non-Cryst. Solids.

[17] Kurdesau F., Khripunov G., da Cunha A., Kaelin M., Tiwari A. (2006). Comparative study of ITO layers deposited by DC and RF magnetron sputtering at room temperature. J. Non-Cryst. Solids.

[18] Bahari A., Sadeghi-Nik A., Shaikh F.U.A., Sadeghi-Nik A., Cerro-Prada E., Mirshafiei E., Roodbari M. (2022). Experimental studies on rheological, mechanical, and microstructure properties of self-compacting concrete containing perovskite nanomaterial. Struct. Concr..

[19] Zhu Y.H., Zhang J.C., Chen Z.T., Egawa T. (2009). Demonstration on GaN-based light-emitting diodes grown on 3C-SiC/Si(111). J. Appl. Phys..

[20] Jin X., Ma B., Zhao K., Zhang Z., Deng J., Luo J., Yuan W. (2020). Effect of annealing on the thermoelectricity of indium tin oxide thin film thermocouples. Ceram. Int..

[21] Kim Y., Park S., Kim S., Kim B.-K., Choi Y., Hwang J.-H., Kim H.J. (2017). Flash lamp annealing of indium tin oxide thin-films deposited on polyimide backplanes. Thin Solid Film..

[22] Park J., Buurma C., Sivananthan S., Kodama R., Gao W., Gessert T. (2014). The effect of post-annealing on Indium Tin Oxide thin films by magnetron sputtering method. Appl. Surf. Sci..

[23] Raoufi D., Kiasatpour A., Fallah H.R., Rozatian A.S.H. (2007). Surface characterization and microstructure of ITO thin films at different annealing temperatures. Appl. Surf. Sci..

[24] Mergel D., Stass W., Ehl G., Barthel D. (2000). Oxygen incorporation in thin films of In_2_O_3_: Sn, prepared by radio frequency sputtering. J. Appl. Phys..

[25] Shi X., Tian Y., Shen C., Wang C., Gao H.J. (2012). Electrodeposition of Sb_2_Se_3_ on indium-doped tin oxides substrate: Nucleation and growth. Appl. Surf. Sci..

[26] Wang C., Liu Y., Xia Y., Ma T., Wang P.W. (2007). Characteristics of ITO films fabricated on glass substrates by high intensity pulsed ion beam method. J. Non-Cryst. Solids.

[27] Ali A.H., Shuhaimi A., Hassan Z. (2014). Structural, optical and electrical characterization of ITO, ITO/Ag and ITO/Ni transparent conductive electrodes. Appl. Surf. Sci..

[28] Han B., Chen L., Jin S., Guo S., Park J., Yoo H.S., Park J.H., Zhao B., Jung Y.M. (2021). Modulating Mechanism of the LSPR and SERS in Ag/ITO Film: Carrier Density Effect. J. Phys. Chem. Lett..

[29] Khachatryan H., Kim D.J., Kim M., Kim H.K. (2018). Roll-to-Roll fabrication of ITO thin film for flexible optoelectronics applications: The role of post-annealing. Mater. Sci. Semicond. Process..

[30] Shajari D., Bahari A., Gill P., Mohseni M. (2017). Synthesis and tuning of gold nanorods with surface plasmon resonance. Opt. Mater..

[31] Bhattacharyya J., Chaudhturi S., Pal A.K. (2020). Studies on the optical properties and the Burstein-moss shift in indium tin oxide films. Phys. Status Solidi.

[32] Fujiwara H., Kondo M. (2005). Effects of carrier concentration on the dielectric function of ZnO: Ga and In2O3: Sn studied by spec-troscopic ellipsometry: Analysis of free-carrier and band-edge absorption. Phys. Rev. B.

